# Control chart for geometrically distributed data based on Bayesian fast double bootstrap

**DOI:** 10.1016/j.mex.2025.103307

**Published:** 2025-04-09

**Authors:** Muhammad Yahya Matdoan, Muhammad Mashuri, Muhammad Ahsan

**Affiliations:** a Department of Statistics, Faculty of Science and Data Analytics, Institut Teknologi Sepuluh Nopember, Kampus ITS-Sukolilo, Surabaya 60111, Indonesia; b Department of Statistics, Faculty of Science and Technology, University of Pattimura, Jl. Ir. M. Putuhena, Kampus Unpatti-Poka, Ambon 97233, Indonesia

**Keywords:** Bayesian fast double bootstrap, g-chart, Minimum variance unbiased, *g*-Chart Using MVU Estimator and BDFB Estimator

## Abstract

Accurate parameter estimation is a critical component of effective process control using g charts. While traditional methods like maximum likelihood and Bayesian estimation are widely used, th ey may exhibit limitations in small sample size scenarios, leading to inaccurate parameter estimates. To address these challenges, minimum variance unbiased (MVU) estimators have been developed. For specific conditions, such as limited data and no nonconforming items, bootstrap-based Bayesian estimators offer a computational alternative. However, these estimators may struggle to detect significant process shifts, particularly in the presence of large deviations. This research introduces a novel Bayesian fast double bootstrap approach for parameter estimation in g-charts. By efficiently handling small sample sizes and effectively detecting large process shifts, this method aims to significantly enhance the accuracy and reliability of process monitoring. The proposed approach leverages the strengths of both bootstrap and double bootstrap techniques, while addressing their limitations through a computationally efficient algorithm. This advancement is expected to contribute to improved process control and quality assurance in various industrial applications. Key points:

• A Bayesian fast double bootstrap (BFDB) approach was developed for parameter estimation in process monitoring, particularly for small sample sizes. Comparative analysis with minimum variance unbiased (MVU) estimators demonstrated the superior sensitivity and computational efficiency of BFDB for process monitoring
• A comparative analysis of BFDB and MVU parameter estimation methods revealed that BFDB consistently outperformed MVU in high-quality process monitoring scenarios.

## Graphical abstract

**Figure:**
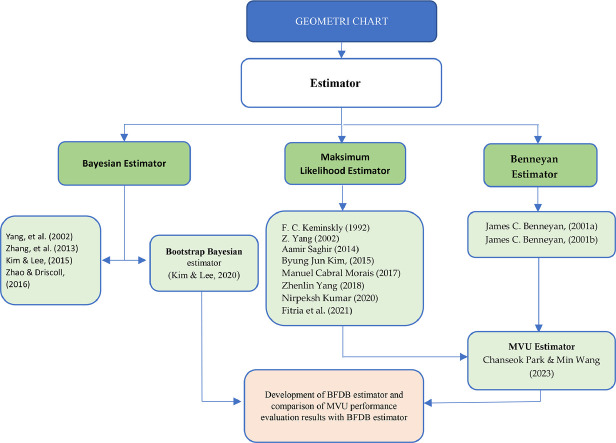

Specifications table

**Table:** 

Subject area:	Mathematics and Statistics
More specific subject area:	Statistics; Statistical Process Control
Name of your method:	g-Chart Using MVU Estimator and BDFB Estimator
Name and reference of original method:	C. Park and M. Wang, “A study on the g and h control charts,” *Commun Stat Theory Methods*, vol. 52, no. 20, pp. 7334–7349, 2023, doi: 10.1080/03,610,926.2022.2044492. B. J. Kim and J. Lee, “Adjustment of Control Limits for Geometric Charts,” *Commun Stat Appl Methods*, vol. 22, no. 5, pp. 519–530, Sep. 2015, doi: 10.5351/csam.2015.22.5.519.
Resource availability:	Share price data for state-owned banks can be accessed through the https://www.idx.co.id/id website and simulation data.

## Background

Control charts are essential tools for monitoring process quality characteristics. They can be broadly classified into two categories: variable control charts and attribute control charts. Variable control charts are employed to monitor continuous data, such as measurements obtained from manufacturing processes. In contrast, attribute control charts are utilized to track discrete data, such as categorical outcomes like conforming or nonconforming products [1]. Commonly employed attribute control charts, such as the n, np, c, and u charts, often prove inadequate for monitoring processes characterized by low defect rates or infrequent events. These limitations can result in a high rate of false alarms or a decreased sensitivity to actual process shifts [2]. To address the challenges of monitoring processes with rare or infrequent nonconforming events, Kaminsky F. C. et al. [3] introduced a geometric distribution based control chart, known as the g-chart. The g-chart is a control chart used to monitor the total number of events. In Addition, The advantage of the g-chart is that it is able to monitor processes with very small or rare conforming events (high quality). Since it was proposed by Kaminsky et al. [3], the g-chart has continued to develop and is widely used from various fields such as monitoring health care [4], monitoring services in banking [5], monitoring plywood product defects [6], and so on.

If the process parameters, on the *g*-chart are unknown, the maximum likelihood (ML) estimator is used [3]. However, the maximum likelihood estimator has the disadvantage that it tends to give a large bias on small sample sizes. In addition, this estimator is less effective for samples with unbalanced subgroup sizes, which can reduce the ability to detect process changes. To overcome these problems, Benneyan J. C [7] developed the Benneyan estimator, but there are still problems with large variance sizes on relatively small data. So [8] developed the minimum variance unbiased (MVU) estimator to overcome these problems in the monitoring process.

The advantages of the MVU estimator on the *g*-chart are that it has a lower variance, in addition, the MVU estimator is more accurate at small sample sizes and can overcome the problem of sample size imbalance (unequal subgroup sizes) so that the resulting control limits are more accurate[9]. In addition, a geometric control chart estimator based on the Bayes estimator has been developed by [[9], [10], [11]], and [11], to overcome the problems with the MVU estimator which is often ineffective, if there are no nonconforming items in the very small phase I sample [12]. This estimator uses a prior distribution to incorporate prior information about the corresponding parameters. However, it still has weaknesses if there are no nonconforming items in a very small phase I sample, so computational estimators such as bootstrap-based Bayesian estimators were developed to overcome these problems [12].

The bootstrap method is a statistical resampling technique that estimates the sampling distribution of a statistic by repeatedly drawing random samples with replacements from the original dataset [13]. The bootstrap method can effectively refine the control limits of geometric charts, even when operating with limited phase I sample sizes, resulting in enhanced accuracy and efficiency [13]. While the bootstrap method can enhance control chart performance, it may exhibit suboptimal sensitivity to larger process shifts [14]. To address these limitations, the double bootstrap method was developed. This technique effectively mitigates bias, narrows confidence intervals, and reduces model estimation errors [[14], [15], [16], [17], [18], [19]]. Thus, double bootstrap is used to improve the efficiency of control charts in monitoring larger shifts [20].

To address the limitations of traditional parameter estimation methods for *g*-charts, the double bootstrap technique was introduced. This approach involves a two-stage resampling process: first, the original dataset is bootstrapped to generate B1 samples; second, each of these B1 samples is further bootstrapped B2 times. This iterative resampling procedure provides a more robust and accurate estimation of the underlying parameter [21]. A significant drawback of the double bootstrap method is its high computational cost due to the iterative resampling process. This can lead to prolonged processing times and increased computational resource requirements, particularly when dealing with large datasets or time constrained analyses [22]. As a solution [23] developed a fast double bootstrap (FDB) which assumes that the first stage bootstrap dataset and the test statistics on the second stage bootstrap dataset are mutually independent, thus for each first stage bootstrap dataset only one replication is performed on the second stage bootstrap. This method produces the same level of accuracy as the double bootstrap method but requires a much shorter processing time to maintain process efficiency [26].

Some recent relevant research in the development of g−charts are [12] on geometric charts with bootstrap based control limits using the bayes estimator. This approach addresses the challenges associated with small sample sizes by enhancing chart accuracy and performance. Park C. et al. [25] proposed robust *g*-type control charts for nonconformity monitoring. Their approach involved incorporating a memoryless estimator and truncation techniques to mitigate the impact of outliers, resulting in a more robust estimator. Furthermore, Park C. et al. [26] proposed a novel approach for constructing robust *g*-charts by leveraging the generalized Kullback-Leibler (GKL) divergence. Their method defines a distance measure between probability distributions based on the GKL divergence, leading to a more robust estimator for the process parameter. This approach offers advantages over memoryless and truncation estimators by mitigating the detrimental effects of outliers on data analysis.

A comprehensive review of existing literature reveals a notable absence of research on *g*-charts constructed using Bayesian estimators based on the fast double bootstrap method. This study addresses this gap by introducing a novel algorithm that integrates Bayesian estimation with fast double bootstrap techniques. The primary objective is to enhance the accuracy and performance of the *g*-chart. To evaluate the effectiveness of the proposed approach, a comparative analysis with the MVU estimator is conducted using the average run length (ARL) metric. ARL serves as a standard measure for assessing control chart performance, representing the average number of observations before the first out-of-control signal is detected [27,28].

## Method details

### g-Chart

The geometric distribution is a distribution that describes the number of trials required for a successful event to occur for the first time. If x number of trials are repeated until the first trial is successful, then x−1 number of failed trials are obtained.

Suppose given data from the process is available as a subgroup or sample of size n, written as $x_{1},\;x_{2},x_{3},\ldots ,x_{n}$. This experiment is a mutually independent and identically distributed experiment from a geometric distribution under controlled or stable conditions. The statistics used to create a control chart is the total number of trials. The random variable for the total number of trials is expressed by the following Eq. (1) [25].

(1) $$T=x_{1}+x_{2}+\cdots +x_{n}$$

so that the mean and variance of each are obtained, namely.

(2) $$\begin{array}{ccc} E\left(T\right) & = & E\left(\sum_{i=1}^{n}x_{i}\right)\\ & = & n\left(\frac{1-p}{p}+a\right)\; \end{array}$$

(3) $$\begin{array}{ccc} V\left(T\right) & = & V\left(\sum_{i=1}^{n}x_{i}\right)\\ & = & \;n\;V\left(X\right) \end{array}$$

### Parameter estimation *g*-Chart

According to Kaminsky et al. [3], if the value of (*p*) in the *g*-chart is unknown, it is necessary to estimate the parameters using the maximum likelihood estimator shown in Eq. (4) below [3].

(4) $${\hat{\;p}}_{ml}=\frac{1}{\bar{\bar{x}}-a+1}$$

When process parameters are unknown, the maximum likelihood estimator is commonly employed for *g*-chart. However, this estimator exhibits a bias for small sample sizes and is less effective for unbalanced subgroups, potentially compromising its ability to detect process shifts. To overcome these problems, [7] developed the Benneyan estimator shown in Eq. (5) below.

(5) $${\hat{P}}_{b}=\frac{\left(N-1\right)/N}{\bar{\bar{x}}-a+1}$$

Despite its widespread use, the Benneyan estimator exhibits a notable limitation in its relatively large variance. To address this shortcoming, [8] introduced the minimum variance unbiased (MVU) estimator. Specifically designed for *g*-charts, the MVU estimator offers a more accurate and efficient estimation of process parameters by minimizing variance. The MVU estimator on the *g*-chart is shown in Eq. (6) below [8].

(6) $${\hat{P}}_{mvu}=\frac{n-1}{\sum_{i=1}^{n}X_{i}-na+n-1}=\frac{\left(n-1\right)/n}{\bar{\bar{x}}-a+1-1/n}$$

The MVU estimator for *g*-chart offers several key advantages. Its minimum variance property ensures efficient parameter estimation. Additionally, the MVU estimator demonstrates superior accuracy, particularly in small sample size scenarios. Furthermore, it effectively addresses the challenge of sample size imbalance, enabling the construction of more precise control limits [8]. Furthermore, the control limits of the MVU estimator based on [8] obtained the upper control limit (UCL) and lower control limit (LCL) as follows.

$$\text{UCL}=n_{k}\bar{\bar{x}}+g\sqrt{\frac{n_{k}N}{N+1}\left(\bar{\bar{x}}-a\right)\left(\bar{\bar{x}}-a+1\right)}$$

$$\text{LCL}=n_{k}\bar{\bar{x}}-g\sqrt{\frac{n_{k}N}{N+1}\left(\bar{\bar{x}}-a\right)\left(\bar{\bar{x}}-a+1\right)}$$

In addition to the conventional estimators (maximum likelihood, benneyann and MVU), estimators have been developed on the *g*-chart based on bayes estimator by [[9], [10], [11]], and [11], to overcome the problem that conventional estimators are often ineffective, if there are no nonconforming items in a very small phase I sample [12].

The Geometric Distribution has a density shown in Eq. (7).

(7) $$f\left(y_{i}|p_{0}\right)={\left(1-p_{0}\right)}^{y_{i}}p_{0};\;y_{i}=0,\;1,\;2,\;\ldots$$

with mean:

$$E\left(y_{i}\right)=\frac{1-p_{0}}{p_{0}}$$

and variance:

$$V\left(y_{i}\right)=\frac{1-p_{0}}{p_{0}^{2}}=\frac{E\left(y_{i}\right)}{p_{0}}$$

The Fisher information of the geometric distribution is shown in Eq. (8).

(8) $$I\left(p_{0}\right)={p_{0}}^{-2}{\left(1-p_{0}\right)}^{-1}$$

The Jeffreys Prior of the geometric distribution is shown in Eq. (9).

(9) $$\pi \left(p_{0}\right)\propto {p_{0}}^{-1}{\left(1-p_{0}\right)}^{-\frac{1}{2}}$$

The posterior $p_{0}$ based on Jeffreys prior is shown in Eq. (10).

(10) $$\pi \left(p_{0}\rfloor yi\right)=Beta\left(N,\;\sum_{i=1}^{n}y_{i}+\frac{1}{2}\right)$$

The mean and variance of the posterior $\pi (p_{0}\rfloor yi)$ can be known, respectively.

(11) $$E_{post}\left(p_{0}\right)=\frac{N}{N+\sum_{i=1}^{N}y_{i}+\frac{1}{2}}$$

(12) $$V_{post}\left(p_{0}\right)=\frac{N\left(\sum_{i=1}^{N}y_{i}+\frac{1}{2}\right)}{{\left(N+\sum_{i=1}^{N}y_{i}+\frac{1}{2}\right)}^{2}\left(N+\sum_{i=1}^{N}y_{i}+\frac{3}{2}\right)}$$

$p_{0}$ is the proportion of nonconforming. Bourke [29] showed that the control limits can be easily determined using the cumulative distribution function of geometric random variables. Specifically, the upper control limit (UCL) and lower control limit (LCL) of the geometric chart must satisfy the following conditions [29]:

(13) $$\sum_{y_{i}=0\;}^{\text{LCL}-1}{\left(1-p_{0}\right)}^{y_{i}}p_{0}=\frac{\alpha }{2}$$

(14) $$\sum_{y_{i}=\text{UCL}+1}^{\infty }{\left(1-p_{0}\right)}^{y_{i}}p_{0}=\frac{\alpha }{2}$$

Where α indicates the desired false alarm rate (FAR). With simple mathematical manipulations, [12], showed that we can easily determine the control limits for the *g*-chart as follows.

$$\hat{UCL}=\frac{\ln\left(\frac{\alpha }{2}\right)}{\ln\left(1-\bar{{\hat{p}}_{0}^{**}}\right)}-1$$

$$\hat{LCL}=\frac{\ln\left(1-\frac{\alpha }{2}\right)}{\ln\left(1-\bar{{\hat{p}}_{0}^{**}}\right)}$$

While the Bayesian estimator incorporates prior information through a prior distribution, it may exhibit limitations in certain scenarios. For instance, when dealing with very small phase I samples and the absence of nonconforming items, computational estimators such as bootstrap-based Bayesian methods offer a promising alternative [12].

Bootstrap method is one of the methods developed by [13], this method is a simulation technique based on data when the sampling distribution of a statistic is unknown or difficult to find. The bootstrap method enhances the accuracy and reliability of *g*-chart control limits, particularly when dealing with limited phase I sample sizes. By generating numerous simulated samples, it effectively reduces ARL variability and mitigates the challenges associated with small sample parameter estimation. This leads to a decrease in false alarms, thereby preserving process efficiency. However, a notable drawback of the bootstrap method lies in its inherent variability, which can result in inconsistent detection outcomes across different observations. This limitation is particularly critical in applications where consistent process monitoring is paramount. To address these shortcomings, the double bootstrap method was developed.

The basic principle of the double bootstrap method is that from the first stage bootstrap result data set of B1, resampling is done again as many as B2 replications of the second stage bootstrap [21]. The weakness of the double bootstrap method is that the calculation time required is longer because it has to calculate as many as B1 + B1xB2 test statistical values [22]. As a solution [23], developed a fast double bootstrap (FDB) which assumes that the first stage bootstrap dataset and the test statistics on the second stage bootstrap dataset are mutually independent, thus for each first stage bootstrap dataset only one replication is performed on the second stage bootstrap. This method produces the same level of accuracy as the double bootstrap method, but requires a relatively short processing time to maintain process efficiency [24,23] introduced the Fast Double Bootstrap (FDB) method as a computationally efficient alternative to the traditional double bootstrap procedure. By leveraging the assumption of independence between test statistics in the first and second bootstrap stages, FDB significantly reduces the required resampling iterations. This computational efficiency translates to substantial time and cost savings without compromising the accuracy of the results.

The Fast Double Bootstrap (FDB) assumes that $\tau_{b^{**}}$​ is independent of $\tau_{b^{*}}$​, so each first-stage bootstrap sample is generated only once in the second-stage bootstrap process. After obtaining the first-stage bootstrap data $X_{b^{*}}$​ and the test statistic $\tau_{b^{*}}$​, where b=1,2,…,B, a second-stage bootstrap sample data is generated for each first-stage bootstrap sample, denoted by $X_{b^{**}}$​ with b=1,2,…,B.

From the set of second-stage bootstrap sample data, the test statistic $\tau_{bj^{**}}$​ can be calculated for b=1,2,…,B. From these results, $Q^{**}\left(1-{\hat{p}}^{*}\right)$ can be calculated, which is the $\left(1-{\hat{p}}^{*}\right)$-th quantile of ${\tau_{b}}^{**}$​ or equivalently, the value of ${\tau_{b}}^{**}$​ at the $(1-{\hat{p}}^{*})B$ -th order. The FDB p-value is calculated using the following Eq. (15).

(15) $${\hat{p}}^{**}=\frac{\text{number}\;\text{of}\;\left({\tau_{b}}^{**}\geq Q^{**}\left(1-{\hat{p}}^{*}\right)\right)}{B}$$

**Fig. 1:**
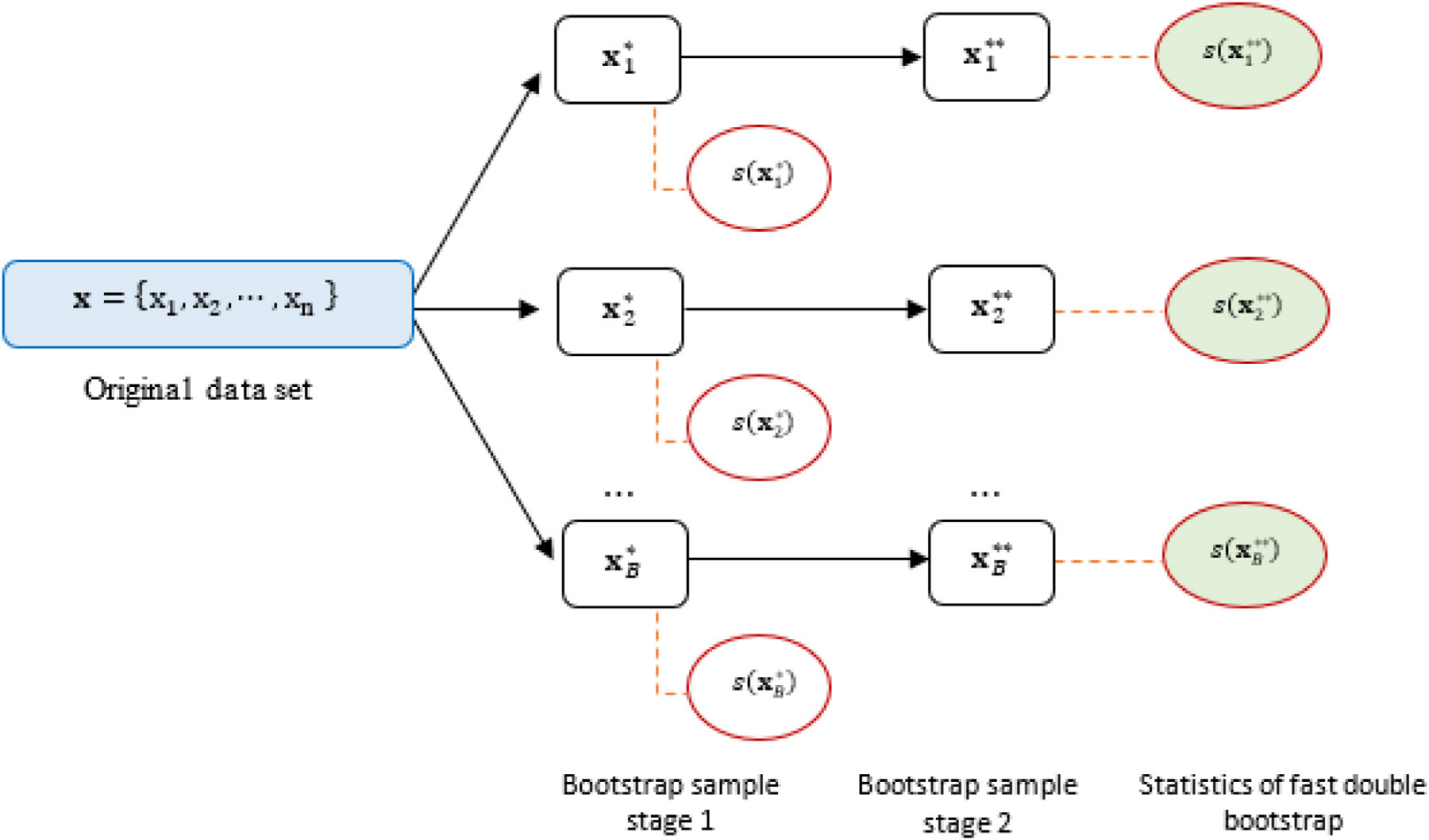
Fast double bootstrap procedure.

Fig. 1 provides a visual representation of the fast double bootstrap procedure, outlining the key steps involved in this computationally efficient resampling technique.

### Method validation

This research procedure for model validation consists of several parts, as follows.

**a. The *g*-chart procedure uses the MVU estimator.**

 1. Determine the number of samples*.*

 2. Calculate UCL and LCL with the following equation.

$$\text{UCL}=n_{k}\bar{\bar{x}}+g\sqrt{\frac{n_{k}N}{N+1}\left(\bar{\bar{x}}-a\right)\left(\bar{\bar{x}}-a+1\right)}$$

$$\text{LCL}=n_{k}\bar{\bar{x}}-g\sqrt{\frac{n_{k}N}{N+1}\left(\bar{\bar{x}}-a\right)\left(\bar{\bar{x}}-a+1\right)}$$

**b. The *g*-chart procedure uses the BFDB estimator.**

 1. Given a set of Prior distributions for $p_{0}\;$based on jeffreys prior i.e. $\pi \left(p_{0}\right)\propto {p_{0}}^{-1}{\left(1-p_{0}\right)}^{-1/2}$ of phase 1. Then estimate the proportion of defects $p_{0}$ using the Bayesian method.

$${\hat{p}}_{0,Bayes}=\frac{N}{m+\frac{1}{2}}$$

 2. Generate as many bootstrap samples as B: $N_{1}^{*},\;N_{2}^{*},\;\ldots ,\;N_{B}^{*}$ of the Binomial distribution $B(m,\;{\hat{p}}_{0,Bayes})$ based on Step 1, and calculate it.

$${\hat{p}}_{0,j}^{*}=\frac{N_{j}^{*}}{m+\frac{1}{2}},\;j=1,\ldots ,B$$

where *B* is a large number.

 3. Based on the results of Stage 1 bootstrap, then in stage 2 bootstrap (fast double bootstrap)

 a. For the first stage bootstrap sample $p_{0,j}^{*}$, perform resampling once to generate a set of second-stage bootstrap data set $N_{j}^{**}$.

 b. Calculate the second stage bootstrap test statistic.

$${\hat{p}}_{0,j}^{**}=\frac{N_{j}^{**}}{m+\frac{1}{2}},\;j=1,\ldots ,B$$

 c. Calculate the quantiles of the distribution ${\hat{p}}_{0,j}^{**}$ at $1-{\hat{p}}^{*}$ confidence level.

$$Q^{**}\left(1-{\hat{p}}^{*}\right)\;\text{dengan}\;{\hat{p}}^{*}=\frac{1}{B}\sum_{j=1}^{B}p_{0,j}^{*}$$

 4. Based on the result of step 3, calculate

$$\bar{{\hat{p}}_{0}^{**}}=\frac{1}{B}\sum_{j=1}^{B}{\hat{p}}_{0,j}^{**}$$

 5. Based on the results of Step 4, construct the UCL, LCL as follows (Zhang et al., 2013).

$$\hat{UCL}=\frac{\ln\left(\frac{\alpha }{2}\right)}{\ln\left(1-\bar{{\hat{p}}_{0}^{**}}\right)}$$

$$\hat{LCL}=\frac{\ln\left(1-\frac{\alpha }{2}\right)}{\ln\left(1-\bar{{\hat{p}}_{0}^{**}}\right)}-1$$

Suppose $X_{i}$ is the number of eligible items between two consecutive ineligible items in phase II with probability of ineligible shift from $p_{0}$ to p [9] defines $C_{i}$ event as:

$$C_{i}=\left\{X_{i}>\hat{UCL}\left(N\right)\vee X_{i}<\hat{LCL}\left(N\right)\right\}$$

Next, calculate ARL as follows.

$$ARL_{*}=E\left(\frac{1}{\alpha \left(N\right)}\right)$$

with

$$\alpha \left(N\right)=P\left(C_{i}|N=n\right)=P\left(X_{i}>\hat{UCL}|N=n\right)+P\left(X_{i}<\hat{LCL}|N=n\right)={\left(1-p\right)}^{\hat{UCL}}-{\left(1-p\right)}^{\hat{LCL}}+1$$

$$E\left(\frac{1}{\alpha \left(N\right)}\right)=\sum_{n=0}^{m}\frac{1}{\alpha \left(n\right)}\left(\begin{array}{c} m\\ n \end{array}\right){p_{0}}^{n}{\left(1-p_{0}\right)}^{m-n}$$

**c. Performance evaluation comparison procedure of MVU estimator and BFDB estimator.**

 1. ARL and SDARL Calculation:

 a. Calculate the Average Run Length (ARL) and Standard Deviation of ARL (SDARL) for both the MVU and BFDB estimators.

 b. ARL measures the average number of observations before a false alarm occurs, while SDARL quantifies the variability in ARL.

 2. Estimator Comparison:

 a. Compare the calculated ARL and SDARL values for the MVU and BFDB estimators.

 b. Determine the superior estimator based on the following criteria:

 • Lower ARL: Indicates better sensitivity to process shifts.

 • Lower SDARL: Suggests more consistent performance.

d. The procedure of applying the *g*-chart based on the MVU and BFDB estimators.

** 1. Data Acquisition:**

 a. This study utilizes secondary financial data pertaining to Bank stock prices publicly available from the Indonesia Stock Exchange (IDX) website (https://www.idx.co.id/id) for the period January 2021 to June 2024.

 b. The specific data selection process should be clarified. Did you focus on a particular set of Bank stocks or a broader index? Specifying the data source further strengthens the scientific rigor.

**2. Control Limit Determination**

 a. This stage involves the estimation of the g-chart control limits using two distinct methodologies:

Minimum Variance Unbiased (MVU) Estimator: This established approach ensures statistically efficient parameter estimates by minimizing variance.

 b. Bayesian Fast Double Bootstrap (BFDB) Estimator: This novel method leverages the strengths of Bayesian estimation and the computational efficiency of the Fast Double Bootstrap technique to provide improved parameter estimates.

3. ***g*-Chart Construction**

 a. Following the control limit determination utilizing both MVU and BFDB estimators, separate g-charts will be constructed.

 b. These charts will visually depict the number of observations (typically days or time intervals) between consecutive non-conforming events (e.g., abnormal stock price fluctuations).

 c. By comparing the plotted data points with the corresponding control limits for each chart (MVU and BFDB), we can assess the process stability and identify potential deviations from the expected pattern.

### Method simulation

A simulation study is conducted to evaluate the performance of the MVU and BFDB estimators in constructing *g*-chart. Geometrically distributed data was generated with varying sample sizes (*n* = 10, 20, 30, 50 for small samples; *n* = 500, 1000, 5000, 10,000 for large samples) and different in-control nonconforming probabilities (*p* = 0.0001, 0.0005, 0.001, 0.05). The resulting g-chart control limits, calculated using both MVU and BFDB estimators, are summarized in Table 1.

**Table 1:** Control limits of the MVU estimator and the BFDB estimator.

p	Method	m							
		10		20		30		50	
		LCL	UCL	LCL	UCL	LCL	UCL	LCL	UCL
0.0001	MVU	0.00	103.85	0.00	155.22	0.00	233.64	0.00	357.4
	BFDB	0.00	66.01	0.00	132.10	0.00	198.18	0.00	330.3
0.0005	MVU	0.00	279.94	0.00	232.74	0.00	232.14	0.00	415.7
	BFDB	0.00	66.01	0.00	132.10	0.00	198.18	0.00	330.3
0.001	MVU	0.00	137.95	0.00	167.39	0.00	221.38	0.00	565.5
	BFDB	0.00	66.01	0.00	132.10	0.00	192.13	0.00	326.9
0.05	MVU	0.00	203.72	0.00	185.88	0.00	260.22	0.00	241.0
	BFDB	0.00	59.75	0.00	101.39	0.00	124.74	0.00	146.6

A comparative analysis of control limits using the BFDB and MVU estimators at *p* = 0.0001, 0.0005, 0.001, and 0.05, as summarized in Table 1, demonstrates the superior sensitivity of BFDB-based control limits in process monitoring. This finding suggests that the BFDB estimator can significantly enhance the performance of *g*-charts, particularly in high-quality process environments. Furthermore, Table 2 presents a comparative summary of the control limits obtained using different resampling sizes (*m* = 500, 1000, 5000, and 10,000).

**Table 2:** Control limits of the MVU estimator and the BFDB estimator.

p	Method	m							
		500		1000		5000		10,000	
		LCL	UCL	LCL	UCL	LCL	UCL	LCL	UCL
0.0001	MVU	0.00	1191.7	0.000	11,816.6	0.000	115,800.9	0.000	116,722
	BFDB	0.00	3204.1	0.338	6474.4	5.017	29,399.2	8.871	48,227
0.0005	MVU	0.00	3308.1	0.000	14,119.7	0.000	23,466.6	0.000	23,223
	BFDB	0.00	3071.8	0.302	6143.9	1.900	14,169.3	2.368	16,454
0.001	MVU	0.00	2816.7	0.000	11,600.0	0.000	11,687.2	0.000	11,672
	BFDB	0.00	2768.7	0.218	5174.49	0.441	6770.2	0.508	7280
0.05	MVU	0.00	224.66	0.000	222.84	0.000	226.6	0.000	225.1
	BFDB	0.00	137.01	0.000	124.20	0.000	130.2	0.000	130.4

A comprehensive analysis of the g-chart control limits derived from MVU and BFDB estimators, based on extensive simulations (*n* = 500, 1000, 5000, 10,000) and varying probability of nonconforming (*p* = 0.0001, 0.0005, 001, 0.05), demonstrates that the BFDB-based control limits exhibit superior sensitivity in monitoring process deviations. The narrower width of the BFDB control limits indicates a heightened likelihood of detecting smaller process shifts, thereby enhancing the overall effectiveness of the g-chart in identifying subtle deviations from the target process.

### Comparison of performance of MVU estimator and BFDB estimator

To comprehensively evaluate the performance of the *g*-chart under various conditions, a comprehensive simulation study was conducted. The simulation involved repeated trials (10,000 iterations) using different sample sizes (*m* = 10, 20, 30, 50, 70, 100 and *m* = 500, 1000, 5000, 10,000) and varying in-control nonconforming probabilities (*p* = 0.0001, 0.0005, 0.001, and 0.05). The objective was to assess the performance of the *g*-chart when utilizing the MVU and BFDB estimators. The MVU estimator was selected to evaluate the *g*-chart's effectiveness in scenarios with limited phase I data, a common challenge in practical process monitoring. However, it's important to note that real-world applications often require larger datasets. Therefore, the simulation encompassed both small and large sample sizes. To maintain a desired false alarm rate (α = 0.0027), the control limits were adjusted accordingly. The simulation results are summarized in Table 3.

**Table 3:** Evaluation of the performance of MVU estimator.

m	Criteria	p			
		0.0001	0.0005	0.001	0.05
10	AARL	212.1363	216.4355	218.5353	230.4267
	SDARL	2.3628	3.2984	4.4721	37.9409
20	AARL	228.8483	230.4738	232.4738	238.3728
	SDARL	3.1892	2.4739	2.4682	5.3728
30	AARL	233.3728	235.4829	236.2849	237.3839
	SDARL	2.4720	3.4782	6.6292	5.7492
50	AARL	232.1243	234.4739	238.8493	234.4393
	SDARL	1.3722	2.0374	3.4729	4.3628
70	AARL	238.9833	241.5108	239.9833	255.7182
	SDARL	3.2849	3.2747	4.2626	5.1293
100	AARL	239.4782	239.7842	240.0122	268.4784
	SDARL	2.3627	4.2526	5.2526	2.3526
500	AARL	238.7384	250.0385	263.4393	350.4785
	SDARL	5.2623	6.3526	6.4526	7.6273
1000	AARL	340.3728	350.2947	353.4738	360.2748
	SDARL	7.4623	7.5623	8.2513	7.2385
5000	AARL	340.4728	355.4728	360.4284	367.2742
	SDARL	7.4276	8.3627	8.4627	8.7363
10,000	AARL	352.4839	356.4892	366.2749	368.4729
	SDARL	10.3722	11.3828	11.3728	11.0130

A comprehensive analysis of the AARL values for the MVU estimator, presented in Table 3, reveals a clear dependence on the probability of nonconforming in the in-control state (p) and the sample size (m). For large values of m, the AARL consistently increases as p0 decreases, approaching a relatively high value of approximately 370 when p is very small. Conversely, when p is large and m is small, the AARL exhibits significantly lower values. These findings suggest that the MVU estimator demonstrates superior performance in high-quality process monitoring scenarios, particularly when the probability of nonconforming items is relatively high and the sample size is moderate.

To comprehensively evaluate the performance of the BFDB estimator, a comprehensive simulation study was conducted. This involved generating 10,000 simulated datasets under various conditions, including small (*m* = 10, 20, 30, 50, 70, 100) and large sample sizes (*m* = 500, 1000, 5000, 10,000). Additionally, different in-control nonconforming probabilities (*p* = 0.0001, 0.0005, 0.001, and 0.05) were considered, with *B* = 1000 bootstrap replications. The primary objective of employing the fast double bootstrap algorithm was to demonstrate its effectiveness in improving g-chart performance, even when working with limited phase I data. However, recognizing the practical need for larger datasets in real-world process monitoring, the simulation study included both small and large sample scenarios. To ensure a consistent false alarm rate of α = 0.0027, the simulation parameters were carefully chosen. The resulting findings are summarized in Table 4.

**Table 4:** Performance evaluation of BFDB estimator.

m	Criteria	p			
		0.0001	0.0005	0.001	0.05
10	AARL	226.4858	226.4858	226.4858	242.2236
	SDARL	0.0000	0.0000	0.0000	37.9409
20	AARL	234.5834	234.5834	234.5834	246.8177
	SDARL	0.0000	0.0000	0.0000	45.5342
30	AARL	237.1467	237.1467	241.8235	248.4753
	SDARL	0.0000	0.0000	18.6992	52.7131
50	AARL	239.1431	239.1431	240.6800	245.0495
	SDARL	0.0000	0.0000	10.8676	49.5246
70	AARL	239.9833	241.5108	239.9833	255.7182
	SDARL	0.0000	10.8006	0.0000	45.8403
100	AARL	240.6072	242.1276	240.6072	278.3304
	SDARL	0.0000	10.7504	0.0000	40.2617
500	AARL	246.2787	252.3070	267.0987	351.1864
	SDARL	18.0773	26.4124	40.4624	4.0347
1000	AARL	344.9107	352.4379	356.6339	362.6723
	SDARL	14.8999	26.3833	46.3399	1.2185
5000	AARL	349.3528	360.6938	362.5849	369.1357
	SDARL	35.9166	48.8180	28.6060	0.0644
10,000	AARL	356.0768	357.8735	367.0254	369.6545
	SDARL	51.2742	32.6482	15.8822	0.0173

**Fig. 2:**
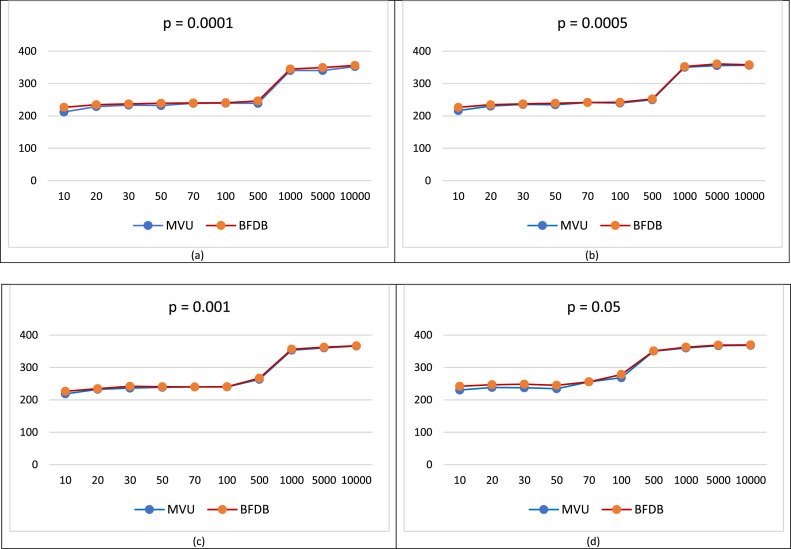
Comparison of performance of MVU estimator and estimator BFDB for *p* = 0.0001 (a), *p* = 0.0005 (b), *p* = 0.001 (c), *p* = 0.05 (d).

A comparative analysis of the BFDB estimator's average run length (ARL) across varying sample sizes (m) and probability of nonconforming in the in-control condition (p) reveals notable trends. For large m and p values, the BFDB estimator exhibits a significantly elevated ARL, approaching 370. Conversely, when m and p are both small, the BFDB estimator demonstrates a relatively low ARL. These findings suggest that the BFDB estimator is particularly effective in detecting deviations from high-quality processes characterized by large m and p values. The visualization of the performance comparison of MVU estimator and BFDB estimator is shown in Fig. 2.

Furthermore, a comprehensive evaluation of the *g*-chart's performance using both MVU and BFDB estimators indicates the superior performance of the BFDB estimator in high-quality process monitoring scenarios. The ARL values presented in Table 3, Table 4 consistently demonstrate the BFDB estimator's ability to detect process shifts more effectively than the MVU estimator, especially when dealing with processes characterized by low nonconforming rates and larger sample sizes.

### Method implementation

The COVID-19 pandemic precipitated a significant disruption across various economic sectors, including the Indonesian banking industry. State-owned banks, such as BRI, Mandiri, BNI, BTN, and BSI, which play a pivotal role in maintaining economic stability, were not immune to the pandemic's adverse effects. The global economic downturn, mobility restrictions, and heightened financial market uncertainty collectively contributed to a decline in the financial performance of these state-owned banks. In response to the pandemic, governments and state banks implemented a range of monetary and fiscal policies aimed at stabilizing the economy. However, the full extent of the pandemic's impact on the banking sector became more evident in the post-pandemic period.

Given that state bank stocks often serve as a barometer of market sentiment regarding financial sector stability, understanding the performance of these emerging bank stocks post-pandemic is crucial. This study seeks to employ a statistical approach, specifically utilizing *g*-charts, to monitor and analyze the performance of state bank stocks. By examining the variability and patterns in stock price fluctuations, g-chart can provide valuable insights into the stability and resilience of the banking sector.

The *g*-chart approach is applied to analyze the nonconforming event, which is defined as the first decline in share price in each month of the study period. The analysis steps include basic parameter calculation, control chart generation, and interpretation. This study uses daily stock price data of state banks obtained from the Indonesian stock exchange (https://www.idx.co.id/id) for the period 2021 to June 2024. The following will discuss the application of the *g*-chart using the data obtained consisting of 42 subgroups. The data is presented in Table 5.

**Table 5:** Empirical data.

Sample *i*	X1	X2	X3	X4	X5	Mean	Total	Sample *i*	X1	X2	X3	X4	X5	Mean	Total
1	2	2	3	2	2	2.2	11	22	3	2	2	3	2	2.4	12
2	2	2	4	3	2	2.6	13	23	2	2	2	2	3	2.2	11
3	2	4	2	5	2	3	15	24	2	5	4	2	2	3	15
4	2	5	4	3	2	3.2	16	25	2	4	3	2	3	2.8	14
5	5	3	5	2	5	4	20	26	2	4	2	2	4	2.8	14
6	3	3	3	2	4	3	15	27	3	3	2	2	2	2.4	12
7	3	2	3	3	2	2.6	13	28	11	2	3	2	5	4.6	23
8	5	6	5	5	2	4.6	23	29	2	2	2	2	6	2.8	14
9	2	7	2	5	2	3.6	18	30	3	2	3	4	2	2.8	14
10	2	3	5	3	3	3.2	16	31	2	2	2	3	3	2.4	12
11	2	2	1	2	2	1.8	9	32	4	4	6	2	2	3.6	18
12	3	3	3	10	2	4.2	21	33	4	4	4	3	2	3.4	17
13	4	3	3	2	3	3	15	34	3	5	3	3	2	3.2	16
14	8	11	2	2	3	5.2	26	35	6	3	5	3	5	4.4	22
15	2	2	2	2	2	2	10	36	3	3	4	3	5	3.6	18
16	2	2	3	3	2	2.4	12	37	2	2	2	2	7	3	15
17	3	2	3	2	3	2.6	13	38	3	6	3	2	3	3.4	17
18	3	3	3	4	3	3.2	16	39	2	10	2	2	2	3.6	18
19	2	2	2	2	2	2	10	40	2	3	2	2	3	2.4	12
20	3	9	4	2	2	4	20	41	4	2	3	2	4	3	15
21	3	2	4	4	2	3	15	42	3	3	3	5	3	3.4	17

### Testing the assumption of geometrically distributed data

Testing the assumption that the data is geometrically distributed aims to determine whether the number of parts that fit between two parts that do not fit follows a geometric distribution or not. This test can be performed using the Kolmogorov-Smirnov test.

Based on Table 6, the Asymp. Sig. (2-tailed) > 0.05, so it can be concluded that the data follows the geometric distribution. Visually, the geometric distribution test can be done using PP-Plot.

**Table 6:** Kolmogorov-Smirnov Test.

Test Statistic	.128
Asymp. Sig. (2-tailed)	.080^c^

**Fig. 3:**
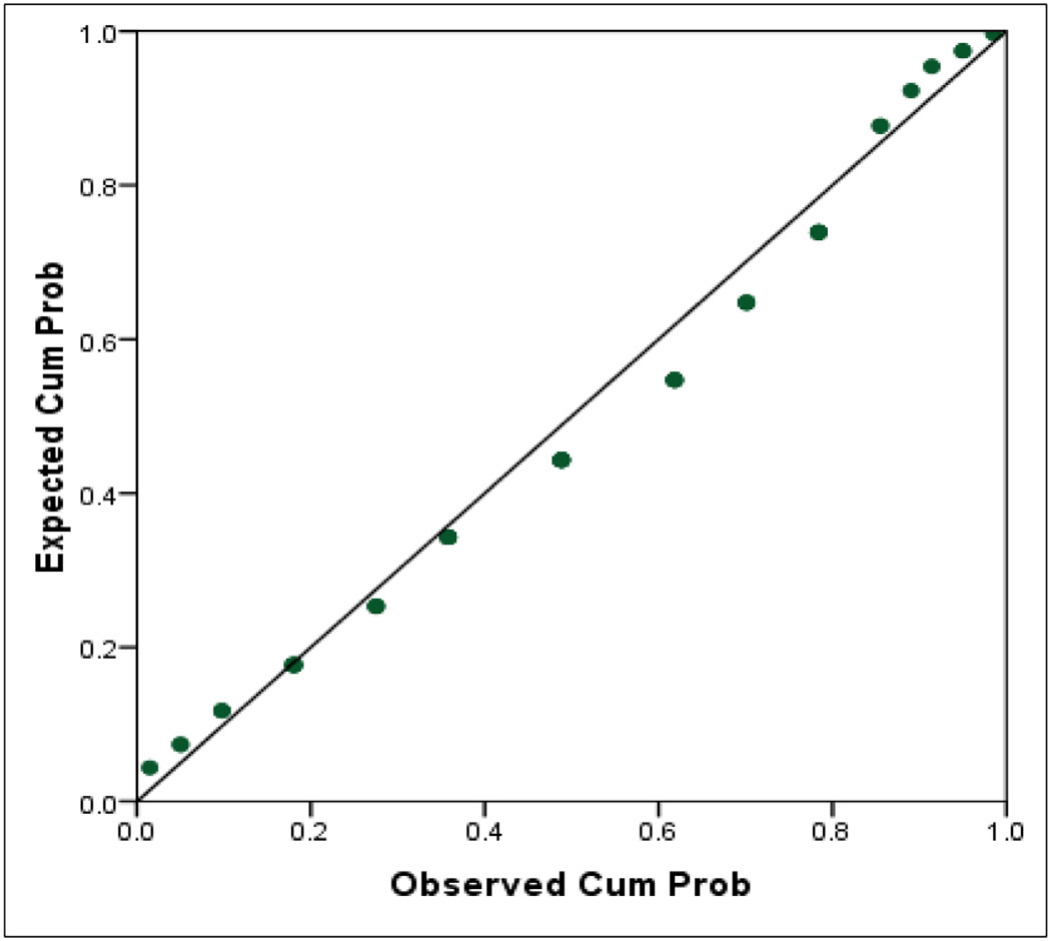
PP-Plot data.

Based on Fig. 3, the data appears to be around a linear line, so it can be concluded that the assumption test is fulfilled because the data in this study follows a geometric distribution.

### *g*-chart using MVU estimator and BFDB estimator

Furthermore Based on Table 5, it is known that the empirical study data used shows the number of increases in the share price of state banks on a certain day of the month until the first decline in the share price. The smallest value (a) in the data is 1 with 42 subgroups (n). Furthermore, to create a *g*-chart using the MVU estimator, it is necessary to calculate ($\bar{\bar{x}}$).

$$\bar{\bar{x}}=\frac{130.6}{42}=3.1$$

Next, calculate CL as follows.

$$\text{CL}=n_{k}\bar{\bar{x}}=15.5$$

Since the value of *a* = 1 and nk=5, then by using *g* = 3, the control limits for the *g*-chart are obtained as follows.

$$\text{UCL}=\left(5\right)\left(3.1\right)+3\sqrt{\frac{\left(5\right)\left(42\right)}{42+1}\left(3.1-1\right)\left(3.1-1+1\right)}=32.5$$

$$\text{LCL}=\left(5\right)\left(3.1\right)-3\sqrt{\frac{\left(5\right)\left(42\right)}{42+1}\left(3.1-1\right)\left(3.1-1+1\right)}=-1.4\;∼0$$

To construct a g-chart utilizing the Bayesian Fast Double Bootstrap (BFDB) estimator, the methodology outlined in Section D of the research procedure should be followed. The resulting parameter estimates and control limits are presented in Table 7.

**Table 7:** Control limits of MVU and BFDB estimators.

Method	Control Limits	
	LCL	UCL
MVU	0	32.5
BFDB	0	30.2

A thorough examination of the control limits derived from the MVU and BFDB estimators, as presented in Table 7, indicates that there are no statistically significant differences between the two approaches. However, a subtle yet noteworthy observation emerges: the BFDB estimator exhibits a slightly heightened sensitivity in process monitoring compared to the MVU estimator. This increased sensitivity suggests that the BFDB estimator may be more effective in detecting subtle deviations from the expected process behavior.

**Fig. 4:**
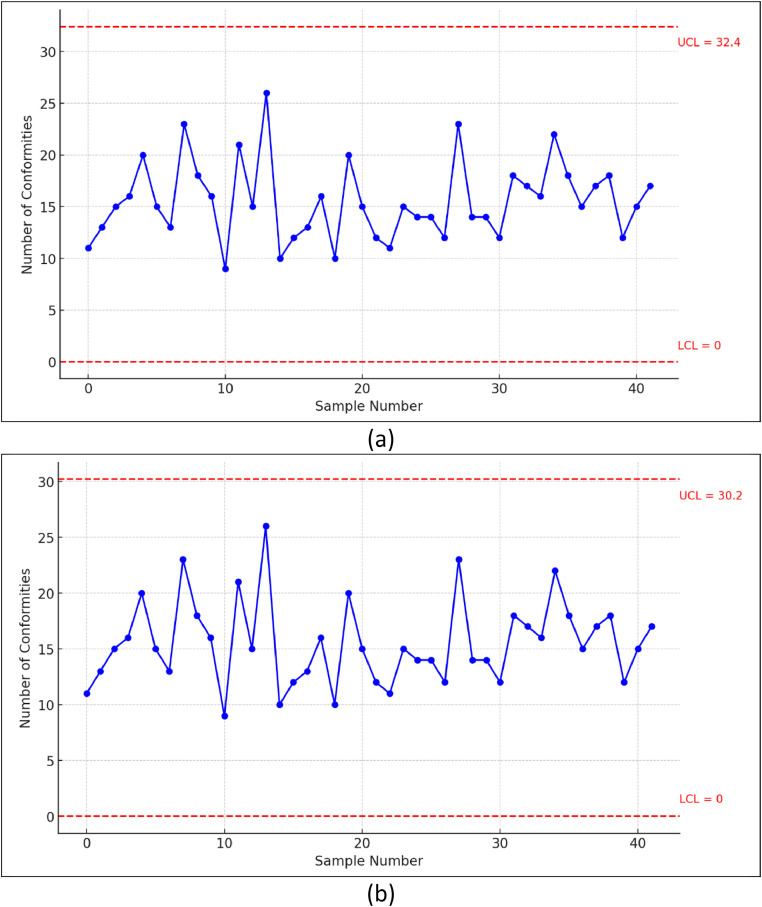
*g*-chart using MVU estimator (a), BFDB estimator (b).

To visually corroborate these findings, Fig. 4 presents a comparative analysis of the g-charts constructed using the MVU and BFDB estimators. This visual representation allows for a direct comparison of the control limits and the plotted data points, providing a clear understanding of the potential differences in sensitivity and detection capabilities between the two approaches.

Fig. 4 presents a visual representation of the g-charts constructed using the Minimum Variance Unbiased (MVU) and Bayesian Fast Double Bootstrap (BFDB) estimators to monitor the stock price behavior of Bank Negara Indonesia during the period January 2021 to June 2024. The analysis reveals that the g-charts, based on both estimators, indicate that the state bank shares remained within the in-control region throughout the study period. This suggests that the stock price process exhibited a stable and predictable pattern. This research offers valuable insights into the post-COVID-19 performance of state bank stocks by leveraging the g-chart methodology. The control charts effectively capture the underlying trends and patterns in stock price fluctuations, providing stakeholders with actionable information. By identifying potential deviations from the expected behavior, these insights enable timely preventive and corrective measures to maintain the stability and long-term performance of state banks in a dynamic and evolving market environment.

The findings of this study demonstrate that the g-chart constructed using the Bayesian Fast Double Bootstrap (BFDB) estimator offers superior performance and sensitivity in process monitoring compared to the traditional Minimum Variance Unbiased (MVU) estimator. Notably, the BFDB approach also exhibits significantly faster data processing times, making it a more efficient choice for practitioners. The application of the g-chart to state bank stock prices during the period 2021-June 2024 revealed that the process remains in a state of control, indicating stable and predictable price behavior. The g-chart utilizing the BFDB estimator can serve as a valuable tool for practitioners to monitor process stability and identify potential deviations in a timely and efficient manner, thereby conserving resources. For future research, it is recommended to explore the development of geometric control charts that are more robust to the presence of outliers. Leveraging bootstrap-based methods could be a promising avenue to enhance the resilience of these charts in the face of data anomalies, ensuring more reliable and accurate process monitoring.

## Limitations

To avoid complexity and wider interpretation, this study identifies the limitation of only determining the parameter estimation of the most sensitive *g*-chart in the monitoring process. The performance measures used in this study only use AARL and SDARL. In addition, the parameter estimation discussed is limited to the MVU estimator and the developed BFDB estimator. For future research, it is recommended to develop g-chart control diagrams related to several research titles such as Trapezoidal cubic fuzzy number Einstein hybrid weighted averaging operators and its application to decision making [30], Aggregation operators on triangular cubic fuzzy numbers and its application to multi-criteria decision making problems [31], Group decision making based on cubic fermatean Einstein fuzzy weighted geometric operator [32], Natural gas based on combined fuzzy TOPSIS technique and entropy [33], and Decision-making problem based on generalized interval-valued bipolar neutrosophic Einstein fuzzy aggregation operator [34].

## Ethics statements

The data used in this study are simulation data and secondary data obtained from the Indonesia Stock Exchange during 2021-June 2024. This data is available upon request or can be accessed directly from the Indonesia Stock Exchange website at https://www.idx.co.id/id.

## CRediT authorship contribution statement

**Muhammad Yahya Matdoan:** Conceptualization, Methodology, Software, Writing – original draft, Visualization. **Muhammad Mashuri:** Conceptualization, Methodology, Writing – review & editing, Validation, Supervision. **Muhammad Ahsan:** Conceptualization, Methodology, Writing – review & editing, Validation, Supervision.

## Declaration of competing interest

The authors declare that they have no known competing financial interests or personal relationships that could have appeared to influence the work reported in this paper.

## Data Availability

Data will be made available on request.
